# De Ritis Ratio (Aspartate Transaminase/Alanine Transaminase) as a Significant Prognostic Factor in Patients Undergoing Radical Cystectomy with Bladder Urothelial Carcinoma: A Propensity Score-Matched Study

**DOI:** 10.1155/2019/6702964

**Published:** 2019-08-27

**Authors:** Hyeong Dong Yuk, Chang Wook Jeong, Cheol Kwak, Hyeon Hoe Kim, Ja Hyeon Ku

**Affiliations:** ^1^Department of Urology, Inje University Sanggye Paik Hospital, Seoul, Republic of Korea; ^2^Department of Urology, Seoul National University Hospital, Seoul, Republic of Korea

## Abstract

**Introduction:**

To investigate the correlation between preoperative De Ritis ratio (aspartate transaminase (AST)/alanine transaminase (ALT)) and postoperative outcome in patients with urothelial cell carcinoma (UC) treated with radical cystectomy.

**Materials and Methods:**

We analyzed the clinical and pathological data of 771 patients who underwent radical cystectomy for bladder UC. Patients were divided into two groups according to the optimal value of AST/ALT ratio. The effect of the AST/ALT ratio was analyzed using the Kaplan–Meier method and Cox regression hazard models for patients' cancer-specific survival (CSS), overall survival (OS), and recurrence-free survival (RFS). In addition, propensity score matching of 1 : 1 was performed between the two groups.

**Results:**

Median follow-up was 84.0 (36–275) months. Mean age was 64.8 ± 10.0 years. According to the receiver operating characteristic (ROC) analysis, the optimal threshold of the AST/ALT ratio was 1.1. In Kaplan–Meier analyses, the high AST/ALT group showed worse outcomes in CSS and OS (all *P* < 0.001). Also, RFS (*P* = 0.001) in the Cox regression models of clinical and pathological parameters was used to predict CSS, OS, and AST/ALT ratio (HR 2.15, 95% CI 1.23-3.73, *P* = 0.007) and pathological T stage (HR 4.80, 95% CI 1.19-19.28, *P* = 0.003). To predict OS and AST/ALT ratio (HR 2.05, 95% CI 1.65–2.56, *P* < 0.001), pathological T stage (HR 2.96, 95% CI 0.57–17.09, *P* = 0.037) and positive lymph node (HR 1.71, 95% CI 1.50–1.91, *P* = 0.021) were determined as independent prognostic factors.

**Conclusion:**

Preoperative AST/ALT ratio could be an independent prognostic factor in patients with UC treated with radical cystectomy.

## 1. Introduction

Bladder cancer is a common cancer in the urinary tract [1] and is the ninth most common cancer worldwide [2]. The most common histopathological type of bladder cancer is urothelial cell carcinoma (UC). In bladder UC diagnosis, 75% of cases are diagnosed as nonmuscle invasive bladder cancer (NMIBC) and 25% are diagnosed as muscle invasive bladder cancer (MIBC) at the time of diagnosis [3–5]. Radical cystectomy is the standard treatment for the highest risk of NMIBC progression and high-risk NMIBC intolerant to intravesical treatment and localized or regionally advanced MIBC [4–6]. However, even if radical cystectomy is performed, the prognosis is poor. Recurrence occurs in more than 30% of patients after radical cystectomy [7], and bladder cancer is the 13th most common cause of cancer deaths [2].

Various biomarkers have been discussed for early diagnosis and prognosis prediction of bladder cancer [8, 9], such as nuclear matrix protein 22, bladder tumor antigen, soluble FAS, fibroblast growth factor receptor 3, methylation biomarkers, and cytokeratin 20 [8]. In addition, there are several mRNA-based biomarker tests such as Cxbladder monitor, XPERT BC, and bladder cancer test [9].

Alanine aminotransferase (ALT) and aspartate aminotransferase (AST) are well-known liver enzymes found in the heart, skeletal muscle, brain, kidney, and red blood cells, in addition to the liver. Because of this characteristic, they are also used as an indicator of other diseases [10, 11]. These enzymes are used as biomarkers that can predict prognosis in several malignancies such as lung, colorectal, pancreas, breast, and kidney [12–16]. Serum ratios of AST and ALT as well as AST and ALT have also been reported to play a role as biomarkers. De Ritis first reported on the serum activity ratio of AST and ALT as an assessment tool for disease in viral hepatitis studies [17]. AST/ALT ratio has also been reported in recent years as a biomarker that can predict prognosis in renal cell carcinoma [18]. It was reported that AST/ALT might be associated with anaerobic glycolysis [19]. This glucose metabolism was also reported to be associated with urothelial carcinoma (UC), and the association of AST/ALT ratio with the prognosis was reported in upper tract urothelial carcinoma (UTUC) [20]. Therefore, the aim of this study was to evaluate the prognostic value of AST/ALT in UC of the bladder in patients with radical cystectomy.

## 2. Materials and Methods

### 2.1. Study Sample

We retrospectively reviewed the medical records of patients who underwent radical cystectomy for bladder urothelial cell cancer at Seoul National University Hospital from 1991 to 2015. T2-T4 or intravesical bacillus Calmette-Guerin (BCG) intolerance T1 high-grade. All patients underwent radical cystectomy with pelvic lymph node dissection. Patients with a short follow-up period of less than 2 years were excluded. Patients with preoperative liver disease, infection, leukocytosis, inflammatory condition, and muscle-related disease were excluded. Six hundred and seventy-one patients were included. The study was approved by the institutional ethical review board (approval code: H-1903-133-1020), and the study protocol and all related content adhered to the guidelines of the Helsinki declaration.

### 2.2. Study Design

Patients were divided into two groups according to the optimal value of the AST/ALT ratio. The optimal value of the AST/ALT ratio was obtained using the receiver operating characteristic (ROC) curve with the highest sensitivity and specificity. The optimum value of AST/ALT thus obtained was 1.1. Patients were divided into two groups based on AST/ALT 1.1. Clinical and pathological information and prognosis of the two groups were compared. In addition, 1 : 1 propensity matching was performed to compensate for the difference between age, sex, BMI, ASA, neoadjuvant chemotherapy, operative type, and diversion type. The patient's clinical and pathological information was reviewed. Clinical and pathological information included age, gender, body mass index (BMI), American Society of Anesthesiologists (ASA) physical status, operative method, urinary diversion type, pathological tumor/lymph nodes/metastasis (TNM) staging, presence of margin positive, carcinoma in situ, lymphovascular invasion (LVI), number of removed lymph nodes, number of positive lymph nodes, neoadjuvant and adjuvant chemotherapy, and adjuvant radiotherapy. Oncologic outcomes data were also collected for recurrence, mortality, and mortality due to cancer.

All patients were admitted to the hospital 2 days before surgery to undergo a 2-day bowel preparation. A preoperative laboratory blood test was performed at admission. Postoperative follow-up was performed according to our hospital protocol as follows. Follow-up was performed every 3 months until 3 years after radical cystectomy, every 6 months for 5 years postoperative, and every year after the first 5 years postoperative. Routine laboratory tests, urine cytology, urine analysis, and cystoscopy were performed at each follow-up after radical cystectomy. In addition, ultrasonography bladder scans for a postvoid urine check were performed at each follow-up in neobladder patients. Computed tomography (CT) and bone scans were performed every year [21].

### 2.3. Statistical Analysis

The analysis of the continuous variables was expressed as a median value and interquartile range (IQR) or mean value and standard deviation (SD) using descriptive statistics. The analysis of nominal variables is expressed as probability (%) using crossover analysis. The primary endpoint was overall survival (OS), and the secondary endpoints were recurrence-free survival (RFS) and cancer-specific survival (CSS). All oncologic outcomes were analyzed using Kaplan–Meier survival analysis and logrank test. Various factors affecting oncologic outcome were analyzed using Cox proportional hazard regression analysis. Additionally, 1 : 1 propensity score matching was performed and perioperative conditions were matched using nonparsimonious multivariate logistic regression. The perioperative conditions included age, gender, BMI, ASA physical status, operation type, diversion type, tumor size, and neoadjuvant chemotherapy. Pathological stage and grade, surgical margin positivity, LVI, carcinoma in situ (CIS), number of removed lymph nodes, number of positive lymph nodes, adjuvant chemotherapy, and radiotherapy were excluded from propensity matching because these variables cannot be used to determine the preoperative condition. A total of 305 patients with high AST/ALT ratios were matched in a 1 : 1 ratio to 466 patients with low AST/ALT ratios using the nearest neighbor method with 0.02 calibration. The propensity score matching was well calibrated and differentiated in most items with a standardized mean difference of less than 0.05.

All statistical tests were performed using IBM SPSS Statistics, version 22.0 (IBM, Armonk, NY, USA), and a *P* value of <0.05 was considered to indicate statistical significance.

## 3. Results

### 3.1. Clinical and Pathological Characteristics of Patients

A total of 771 patients diagnosed with UC in the bladder who underwent radical cystectomy were included. The median follow-up was 84 months (IQR 36–275). Of the total patients, 84.5% were men and the mean age was 64.8 years (SD ± 10.2). Most patients (91.9%) received open radical cystectomy.  Table 1 shows the clinical and pathological characteristics of the patients. Patients were divided into two groups based on an AST/ALT ratio of 1 : 1. The mean age of the high AST/ALT group was significantly higher than that of the low AST/ALT group (*P* < 0.001). In addition, the tumor size was larger (*P* = 0.017) in the high AST/ALT group. In the pathological T stage, T2 was relatively low in the AST/ALT group, while T3 and T4 were high in the AST/ALT group. In the pathological T stage, T2 was relatively higher in the low AST/ALT group. However, T3 and T4 were higher in the high AST/ALT group (*P* = 0.002). The surgical margin positive rate was higher in the high AST/ALT group (*P* = 0.036), and the number of removed lymph nodes was higher in the low AST/ALT group (*P* = 0.018). The recurrence rate in the high AST/ALT group was higher than that in the low AST/ALT group (*P* = 0.037). Overall, bladder cancer-caused mortality was also higher in the high AST/ALT group than in the low AST/ALT group (*P* < 0.001). After the propensity score matching, 1 : 1 matching was performed for age, gender, BMI, ASA physical status, operation type, diversion type, tumor size, removed lymph node, and neoadjuvant chemotherapy. The propensity score matching was 1 : 1 matched for age, gender, BMI, ASA physical status, operation type, diversion type, tumor size, and neoadjuvant chemotherapy. The propensity score matching was well calibrated and differentiated in most items. However, the mean age and gender were not well matched because of the limited number of populations.

### 3.2. Correlation between Serum Preoperative AST/ALT Ratio and Oncologic and Survival Outcomes before Propensity Score Matching

**Figure 1:**
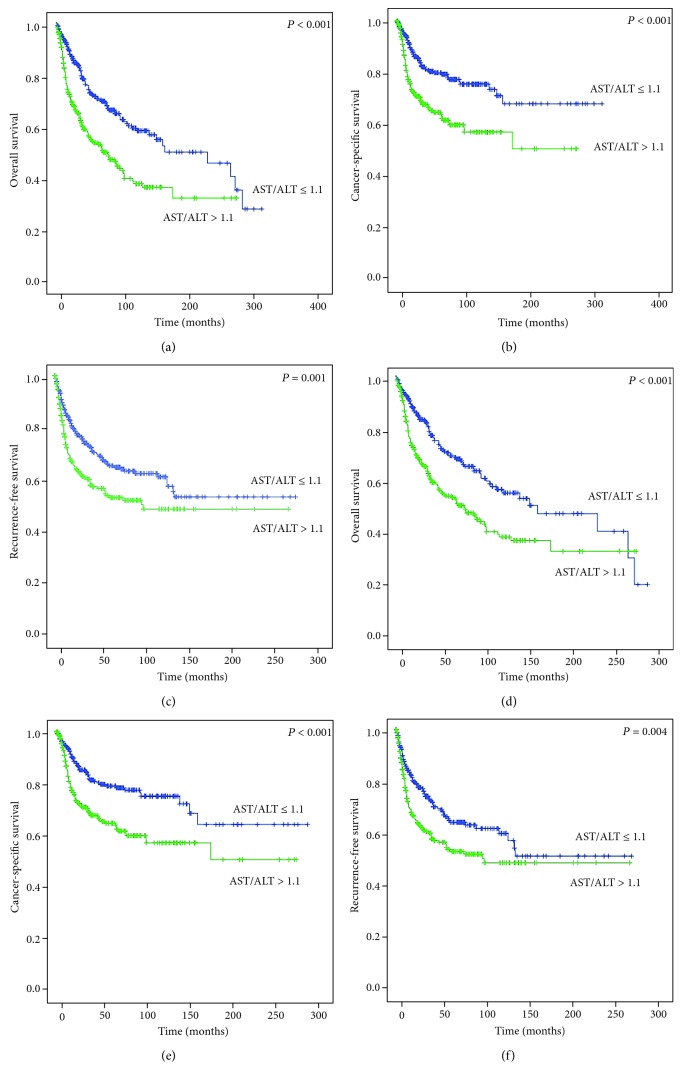
Kaplan–Meier survival curves of overall survival, cancer-specific survival, and recurrence-free survival according to the preoperative aspartate transaminase (AST)/alanine transaminase (ALT) ratio before (a–c) and after (d–f) propensity score matching.

The overall mortality rate was significantly higher (45.9%) in the high AST/ALT group (*P* < 0.001) than in the low AST/ALT group (30.3%). The cancer-causing mortality rate and recurrence rate were also significantly higher in the high AST/ALT group (*P* < 0.001 and *P* = 0.037, respectively) (Table 1). Kaplan–Meier analysis shows that the low AST/ALT group has a better prognosis for OS (*P* < 0.001), CSS (*P* < 0.001), and RFS (*P* = 0.001) than the high AST/ALT group (Figures 1(a)–1(c)). The multivariate Cox analysis showed that a high preoperative AST/ALT ratio was a significant independent predictor of poor prognosis such as OS (HR 2.05, 95% CI 1.65-2.56, *P* = 0.007) and CSS (HR 1.32, 95% CI 0.69-2.56, *P* < 0.001) (Table 2). In addition, the tumor size, high tumor grade, and pathological T and N stages are associated with poor prognosis (Table 2).

### 3.3. After Propensity Score Matching

The overall mortality rate was significantly higher (45.9%) in the high AST/ALT group (*P* < 0.001) than in the low AST/ALT group (31.5%). The cancer-causing mortality rate and recurrence rate were also significantly higher in the high AST/ALT group (*P* = 0.001 and *P* < 0.001, respectively) (Table 1). Kaplan–Meier analysis shows that the low AST/ALT group has a better prognosis for OS (*P* < 0.001), CSS (*P* < 0.001), and RFS (*P* = 0.001) than the high AST/ALT group (Figures 1(d)–1(f)). The multivariate Cox analysis showed that a high preoperative AST/ALT ratio was a significant independent predictor of poor prognosis such as OS (HR 1.57, 95% CI 1.20-2.06, *P* = 0.001), CSS (HR 1.76, 95% CI 1.26-2.48, *P* = 0.001), and RFS (HR 1.53, 95% CI 1.15-2.05, *P* = 0.004) (Table 3). In addition, the tumor size, high tumor grade, and pathological T, N, and M stages are also associated with poor prognosis. The NACH was associated with good prognosis (Table 3).

## 4. Discussion

In our present study, patients with high AST/ALT ratio showed significant association with poor prognosis in clinical outcomes. We analyzed patients who underwent radical cystectomy for MIBC or high-risk bladder cancer intolerant to intravesical BCG treatment. The number of patients was 617 in 24 years. The cohort was relatively large, and the follow-up period was relatively long. We performed a 1 : 1 propensity matching of preoperative factors, except for the AST/ALT ratio to compensate for the bias due to preoperative factors. Due to the limited number of patients, one-to-many propensity matching could not be performed. Propensity matching was not satisfactory, but preoperative factors except mean age and gender matched. Increased AST/ALT ratio before radical cystectomy is a negatively prognostic factor. Preoperative high AST/ALT group has a poor prognosis for OS (*P* < 0.001), CSS (*P* < 0.001), and RFS (*P* = 0.004) than the low AST/ALT group. And preoperative AST/ALT ratio is a significant prognostic factor of postoperative oncologic outcomes. The matched results also show that a preoperative high AST/ALT ratio is negatively correlated with survival outcomes such as OS (HR 1.57, 95% CI 1.20–2.06, *P* = 0.001), CSS (HR 1.76, 95% CI 1.26–2.48, *P* = 0.001), and RFS (HR 1.53, 95% CI 1.15–2.05, *P* = 0.004).

AST/ALT ratio has been reported as a predictor of prognosis in lung cancer, colorectal cancer, pancreatic cancer, breast cancer, and kidney cancer [12–16]. Rawson and Peto conducted a retrospective analysis of 3873 patients with small cell lung cancer. The AST and AST/ALT ratios were considered to be important prognostic indexes [15]. Stoken et al. analyzed the prognostic factors of pancreatic cancer in 653 patients and reported the effect of AST as a prognostic factor [16]. Bezan et al. analyzed 698 patients retrospectively. They reported that the AST/ALT ratio is an independent prognostic factor related to poor prognosis of metastasis-free survival (HR 1.61, 95% CI 1.25-2.07, *P* < 0.001) and OS (HR 1.76, 95% CI 1.34-2.32, *P* < 0.001) in patients with nonmetastatic renal cell carcinoma [18]. Lee et al. analyzed retrospectively 2965 patients with nonmetastatic renal cell carcinoma (RCC). The AST/ALT ratio of 1 : 2 or more was the predictor of poor prognosis of disease progression (HR 1.37, 95% CI 1.00–1.88, *P* = 0.048), overall mortality (HR 1.56, 95% CI 1.07–2.27, *P* = 0.021), and cancer-specific mortality (HR 1.97, 95% CI 1.25–3.12, *P* = 0.004) [22]. In Chougule's study, 92 neck cancer patients without liver metastases and 71 uterine cervix cancer patients had increased AST and ALT values from 133% to 229% of normal value, which decreased to a normal level after radiotherapy [23]. In the O'Reilly et al. study of 312 breast cancer patients, 84% of the patients had biochemical abnormalities in AST, and the elevation levels of AST were associated with an important association of survival (*P* < 0.001) [24]. In the case of UC, Lee et al. retrospectively analyzed 623 UTUC patients who underwent nephroureterectomy [20]. Elevated preoperative AST/ALT is a poor prognostic factor for the postoperative survival outcome of UTUC. The postoperative survival outcomes were PFS (HR 2.33, 95% CI 1.63-3.34, *P* < 0.001), CSS (HR 2.55, CI 1.69-3.85; *P* < 0.001), and OS (HR 2.07, 95% CI 1.41-3.03, *P* < 0.001). Gorgel et al. retrospectively analyzed 153 patients who underwent radical cystectomy [25]. The preoperative AST/ALT ratio was a significant prognostic factor for OS (HR 2.61, 95% CI 1.49-4.56, *P* < 0.001) and disease-free survival (HR 5.79, 95% CI 2.25-15.13, *P* < 0.001). Pathological T stage and age were correlated with prognosis. In addition, the AST/ALT ratio cutoff value was 1.3. In our study, the size of the tumor, neoadjuvant chemotherapy, and type of diversion were correlated with prognosis. The AST/ALT ratio cutoff value was 1.1. Ha et al. retrospectively analyzed 118 patients who underwent radical cystectomy. A high AST/ALT ratio was a poor prognostic factor for metastasis-free survival (HR 2.39, 95% CI 1.16-4.91, *P* = 0.018), CSS (HR 2.75, 95% CI 1.21-6.25, *P* = 0.015), and OS (HR 2.76, 95% CI 1.26-6.07, *P* = 0.011). The AST/ALT ratio cutoff value was 1.3 [26].

Several hypotheses have been presented to explain the association of the AST/ALT ratio with cancer. The most well-known of these hypotheses is the “Warburg effect.” In cancer cell metabolism, glucose uptake and anaerobic glycolysis are increased for the ATP production of adenosine triphosphatase (ATP) [27]. Cancer cells produce sufficient ATP through glycolysis metabolism, thereby promoting the multiplying of cancer cells. In addition, increased glycolysis reduces pH and increases lactate secretion. Reduced pH affects the tumor microenvironment and has a favorable effect on cancer progression, metastasis, and local invasion [28, 29]. Also, increased lactate has been suggested to play an important role in maintaining glycolysis, affecting the lactate dehydrogenase and nicotinamide adenine dinucleotide (NADH)/NAD+ ratio, and affecting the glucose transporter [30]. The AST plays an important role in glycolysis by relocating NADH to mitochondria.

UC is known to be associated with glucose metabolism [31, 32]. In addition, Whyard et al. reported fluorescence microscopy to show the difference in glucose consumption between urothelium and malignant urothelial cells [32] Therefore, although AST and ALT are associated with UC, the mechanism of the association between the UC and AST/ALT ratios is still unclear and further research is needed.

The present study has several limitations. Retrospective research design can have an inherent bias. In addition, the undetectable illness, the condition of the patient, and medications currently taken by the patient may have affected AST and ALT. Finally, our target population was confined to Korean patients and could therefore not reflect the differences among patients according to race. Further research and prospective studies are needed to verify our results.

## 5. Conclusions

Increased AST/ALT ratio before radical cystectomy is a negatively prognostic factor that has a significant effect on the postoperative survival of bladder UC patients. Additional prospective studies are needed to identify the specific mechanisms for bladder UC and AST/ALT ratio.

## Figures and Tables

**Table 1:** Clinicopathological characteristics of patients before and after propensity score matching.

Variables	Before propensity score matching			After propensity score matching		
	Low AST/ALT (*N* = 466)	High AST/ALT (*N* = 305)	*P* value	Low AST/ALT (*N* = 305)	High AST/ALT (*N* = 305)	*P* value
Mean age (year)	62.9 ± 9.9	67.2 ± 10.0	<0.001	64.6 ± 8.8	67.2 ± 10.0	0.001
Gender			<0.001			<0.001
Female	45 (9.6%)	74 (24.3%)		31 (10.2%)	74 (24.3%)	
Male	421 (90.4%)	231 (75.7%)		274 (89.8%)	231 (75.7%)	
BMI (kg/m^2^)	23.7 ± 3.8	22.2 ± 2.9	<0.001	23.0 ± 2.7	22.2 ± 2.9	0.067
ASA			0.373			0.714
1	172 (36.9%)	103 (33.7%)		110 (36.0%)	103 (33.7%)	
2	275 (59.0%)	182 (59.7%)		180 (59.0%)	182 (59.7%)	
≥3	19 (4.1%)	20 (6.6%)		15 (4.9%)	20 (6.6%)	
Operative type			0.218			0.273
Open	424 (91.0%)	285 (93.5%)		295 (93.4%)	285 (93.5%)	
Laparoscopic	24 (5.2%)	16 (5.2%)		11 (3.6%)	16 (5.2%)	
Robot	18 (3.8%)	4 (1.3%)		9 (3.0%)	4 (1.3%)	
Diversion type			0.052			0.168
Conduit	253 (54.3%)	194 (63.6%)		209 (68.5%)	194 (63.6%)	
Neobladder	213 (45.7%)	111 (36.4%)		96 (31.5%)	111 (36.4%)	
Tumor grade			0.396			0.569
Low	12 (2.6%)	11 (3.6%)		10 (3.3%)	11 (3.6%)	
High	454 (97.4%)	294 (96.4%)		295 (96.7%)	294 (96.4%)	
Tumor size (cm)	2.4 ± 3.0	3.3 ± 3.2	0.017	1.2 ± 2.4	3.3 ± 3.2	0.058
Pathological T stage			0.002			0.112
T1	73 (15.7%)	57 (18.7%)		53 (17.4%)	57 (18.7%)	
T2	264 (56.7%)	122 (40.0%)		156 (51.0%)	122 (40.1%)	
T3	106 (22.7%)	104 (34.1%)		80 (26.2%)	104 (34.1%)	
T4	23 (4.9%)	22 (7.2%)		16 (5.2%)	22 (7.2%)	
Margin positive	7 (1.5%)	15 (4.9%)	0.036	5 (1.6%)	15 (4.9%)	0.041
LVI	129 (27.6%)	106 (34.8%)	0.055	90 (29.5%)	106 (34.8%)	0.193
CIS	155 (33.3%)	97 (31.8%)	0.735	97 (31.8%)	97 (31.8%)	1.000
Pathological N stage			0.455			0.544
N0	382 (82.0%)	240 (78.7%)		252 (82.6%)	240 (78.7%)	
N1	34 (7.3%)	20 (6.6%)		20 (6.6%)	20 (6.6%)	
N2	42 (9.0%)	38 (12.5%)		28 (9.2%)	38 (12.5%)	
N3	8 (1.7%)	7 (2.3%)		5 (1.6%)	7 (2.3%)	
Removed LN	18.9 ± 11.7	16.9 ± 10.9	0.018	17.1 ± 11.5	16.9 ± 10.9	0.158
Positive LN	0.7 ± 2.5	1.0 ± 3.0	0.199	0.6 ± 2.3	1.0 ± 3.0	0.146
Pathological M stage			0.749			0.966
M0	462 (99.2%)	304 (99.7%)		363 (99.2%)	304 (99.7%)	
M1	4 (0.8%)	1 (0.3%)		2 (0.7%)	1 (0.3%)	
NACH	64 (13.7%)	39 (12.8%)	0.827	40 (13.1%)	39 (12.8%)	0.976
ACH	99 (21.2%)	74 (24.3%)	0.414	65 (21.3%)	74 (24.3%)	0.440
ART	4 (0.8%)	3 (1.0%)	0.986	3 (1.0%)	3 (1.0%)	1.000
Recurrence rate	139 (29.8%)	115 (37.7%)	0.037	89 (29.2%)	115 (37.7%)	0.032
Mortality	141 (30.3%)	140 (45.9%)	<0.001	96 (31.5%)	140 (45.9%)	<0.001
Cancer-caused mortality	88 (18.9%)	96 (31.5%)	<0.001	59 (19.3%)	96 (31.5%)	0.001
AST/ALT ratio	0.9 ± 0.2	1.7 ± 1.6	<0.001	0.9 ± 0.2	1.7 ± 1.6	<0.001

ASA: American Society of Anesthesiologists; BMI: body mass index; NACH: neoadjuvant chemotherapy; ALT: alanine aminotransferase; AST: aspartate aminotransferase; LVI: lymphovascular invasion; CIS: carcinoma in situ; LN: lymph node; NACH: neoadjuvant chemotherapy; ACH: adjuvant chemotherapy; ART: adjuvant radiotherapy.

**Table 2:** Multivariate Cox proportional hazards analyses of AST/ALT ratio on overall survival, cancer-specific survival, and recurrence-free survival.

Variables	Overall survival		Cancer-specific survival		Recurrence-free survival	
	HR (95% CI)	*P* value	HR (95% CI)	*P* value	HR (95% CI)	*P* value
Age	1.03 (1.00-1.01)	0.032	1.05 (1.01-1.09)	0.022	1.01 (0.97-1.04)	0.633
BMI	0.92 (0.84-1.01)	0.099	0.92 (0.84-1.02)	0.103	1.06 (1.00-1.13)	0.043
Tumor size	1.16 (1.09-1.24)	<0.001	1.16 (1.09-1.24)	<0.001	1.13 (1.07-1.20)	<0.001
High tumor grade	1.58 (1.10-2.28)	0.013	1.65 (1.09-2.49)	0.017	1.30 (0.90-1.88)	0.159
≥pT2 stage	3.30 (1.18-9.21)	0.022	5.78 (2.51-13.35)	<0.001	5.14 (2.05-12.90)	<0.001
≥N1 stage	1.37 (1.06-1.77)	0.014	1.35 (1.05-1.74)	0.018	1.54 (1.20-1.97)	<0.001
≥M1 stage	1.68 (0.23-11.99)	0.605	3.89 (0.45-33.90)	0.219	1.73 (0.22-13.58)	0.604
NACH	1.21 (0.60-2.42)	0.594	1.33 (0.63-2.78)	0.191	1.86 (0.80-4.26)	0.142
AST/ALT ratio						
AST/ALT ≤1.1	Reference		Reference		Reference	
AST/ALT >1.1	2.15 (1.23-3.73)	0.007	2.05 (1.65-2.56)	<0.001	1.32 (0.69-2.56)	0.087

BMI: body mass index; NACH: neoadjuvant chemotherapy; ALT: alanine aminotransferase; AST: aspartate aminotransferase.

**Table 3:** Multivariate Cox proportional hazards analyses of AST/ALT ratio on overall survival, cancer-specific survival, and recurrence-free survival after propensity score matching.

Variables	Overall survival		Cancer-specific survival		Recurrence-free survival	
	HR (95% CI)	*P* value	HR (95% CI)	*P* value	HR (95% CI)	*P* value
Age	1.03 (1.01-1.04)	<0.001	1.02 (0.99-1.03)	0.092	1.00 (0.99-1.02)	0.876
BMI	0.92 (0.87-0.96)	<0.001	0.94 (0.88-0.99)	0.034	0.97 (0.92-1.02)	0.299
Tumor size	1.09 (1.05-1.15)	<0.001	1.11 (1.05-1.17)	<0.001	1.09 (1.04-1.14)	0.001
High tumor grade	1.51 (1.04-2.18)	0.018	1.70 (1.13-2.55)	0.010	1.37 (0.95-1.98)	0.093
≥pT2 stage	5.36 (2.12-13.51)	<0.001	6.99 (3.00-16.28)	<0.001	13.47 (4.25-42.65)	<0.001
≥N1 stage	1.36 (1.05-1.75)	0.018	1.35 (1.05-1.74)	0.018	1.60 (1.25-2.06)	0.002
≥M1 stage	1.27 (0.74-2.18)	0.394	1.71 (0.98-3.00)	0.060	2.10 (1.23-3.60)	0.007
NACH	0.51 (0.35-0.75)	0.001	0.45 (0.29-0.69)	<0.001	0.47 (0.32-0.67)	<0.001
AST/ALT ratio						
AST/ALT ≤1.1	Reference	Reference	Reference	Reference	Reference	Reference
AST/ALT >1.1	1.57 (1.20-2.06)	0.001	1.76 (1.26-2.48)	0.001	1.53 (1.15-2.05)	0.004

BMI: body mass index; NACH: neoadjuvant chemotherapy; ALT: alanine aminotransferase; AST: aspartate aminotransferase.

## Data Availability

The data used to support the findings of this study are available from the corresponding author upon request.
